# Supplementary material for: A molecular dynamics simulation study of the ACE2 receptor with screened natural inhibitors to identify novel drug candidate against COVID-19
Source: PeerJ. 2021 Apr 23;9:e11171. doi: 10.7717/peerj.11171 (PMC8074842; doi:10.7717/peerj.11171)
Supplement: Supplemental Information 1 [file peerj-09-11171-s001.docx]

**Raw Data File**

**Receptor:**

ACE2 human receptor file was downloaded from NCBI having Accession ID Q9BYF1.2.

**Ligand:**

**List of natural compounds along with their PubChem ID pubche id downloaded from Pubchem database**

| **S.No.** | **Compound** | **PubChem ID** |
| --- | --- | --- |
| **1.** | **Choline** | 305 |
| **2.** | **Harmine** | 5280953 |
| **3.** | **Cinnamaldehyde** | 637511 |
| **4.** | **Cinnamic Acid** | 444539 |
| **5.** | **Coumarins** | 54678486 |
| **6.** | **Ursolic Acid** | 64945 |
| **7.** | **Chlorogenic Acid** | 1794427 |
| **8.** | **Assafoetidnol B** | 636584 |
| **9.** | **Glucobrassicin** | 656506 |
| **10.** | **Linamarin** | 11128 |
| **11.** | **Luteolin** | 5280445 |
| **12.** | **Andrographolide (Kalmegh)** | 5318517 |
| **13.** | **Catechin** | 9064 |
| **14.** | **Citral** | 638011 |
| **15.** | **Hydroxychloroquine** | 3652 |
| **16.** | **Linalool** | 6549 |
| **17.** | **Nicotine** | 89594 |
| **18.** | **Chloroquine** | 2719 |
| **19.** | **Allium** | 131751689 |
| **20.** | **Thymoquinone** | 10281 |

**List of synthetic compounds along with their PubChem ID**

| **S.No.** | **Compound** | **PubChem ID** |
| --- | --- | --- |
| **1.** | **Amprenavir** | 65016 |
| **2.** | **Acyclovir** | 135398513 |
| **3.** | **Umifenovir** | 131411 |
| **4.** | **Combivir** | 72187 |
| **5.** | **Tamiflu** | 78000 |
| **6.** | **Zanamivir** | 60855 |
| **7.** | **Cidofovir** | 60613 |
| **8.** | **Peramivir** | 154234 |
